# Effects of *Eimeria tenella* infection on chicken caecal microbiome diversity, exploring variation associated with severity of pathology

**DOI:** 10.1371/journal.pone.0184890

**Published:** 2017-09-21

**Authors:** Sarah E. Macdonald, Matthew J. Nolan, Kimberley Harman, Kay Boulton, David A. Hume, Fiona M. Tomley, Richard A. Stabler, Damer P. Blake

**Affiliations:** 1 Department of Pathobiology and Population Sciences, Royal Veterinary College, University of London, Hatfield, United Kingdom; 2 The Roslin Institute and Royal (Dick) School of Veterinary Studies, University of Edinburgh, Midlothian, United Kingdom; 3 Department of Pathogen Molecular Biology, London School of Hygiene and Tropical Medicine, London, United Kingdom; Universita degli Studi di Camerino, ITALY

## Abstract

*Eimeria* species cause the intestinal disease coccidiosis, most notably in poultry. While the direct impact of coccidiosis on animal health and welfare is clear, its influence on the enteric microbiota and by-stander effects on chicken health and production remains largely unknown, with the possible exception of *Clostridium perfringens* (necrotic enteritis). This study evaluated the composition and structure of the caecal microbiome in the presence or absence of a defined *Eimeria tenella* challenge infection in Cobb500 broiler chickens using 16S rRNA amplicon sequencing. The severity of clinical coccidiosis in individual chickens was quantified by caecal lesion scoring and microbial changes associated with different lesion scores identified. Following *E*. *tenella* infection the diversity of taxa within the caecal microbiome remained largely stable. However, infection induced significant changes in the abundance of some microbial taxa. The greatest changes were detected in birds displaying severe caecal pathology; taxa belonging to the order Enterobacteriaceae were increased, while taxa from Bacillales and Lactobacillales were decreased with the changes correlated with lesion severity. Significantly different profiles were also detected in infected birds which remained asymptomatic (lesion score 0), with taxa belonging to the genera *Bacteroides* decreased and *Lactobacillus* increased. Many differential taxa from the order Clostridiales were identified, with some increasing and others decreasing in abundance in *Eimeria*-infected animals. The results support the view that caecal microbiome dysbiosis associated with *Eimeria* infection contributes to disease pathology, and could be a target for intervention to mitigate the impact of coccidiosis on poultry productivity and welfare. This work highlights that *E*. *tenella* infection has a significant impact on the abundance of some caecal bacteria with notable differences detected between lesion score categories emphasising the importance of accounting for differences in caecal lesions when investigating the relationship between *E*. *tenella* and the poultry intestinal microbiome.

## Introduction

Over the last 20 years global poultry production has tripled with approximately 90 million tonnes of chicken meat and 1.1 trillion eggs now produced every year (http://www.fao.org/faostat/) [1]. Further global expansion is predicted, most notably in Africa and Asia [2], emphasising the importance to food security of effective control against poultry pathogens including the protozoan *Eimeria* species. Members of the phylum Apicomplexa, these parasites can cause the intestinal disease coccidiosis in many animals including poultry. Seven species specifically infect the domestic chicken (*Gallus gallus domesticus*) causing malabsorptive (*Eimeria acervulina*, *E*. *maxima*, *E*. *mitis*, *E*. *praecox*) or haemorrhagic (*E*. *brunetti*, *E*. *necatrix*, *E*. *tenella*) enteritis, with species-specific sites of development and foci of pathology within the intestinal tract [3]. Three species, *E*. *acervulina*, *E*. *maxima* and *E*. *tenella* are most frequently found in intensively reared chickens and the latter is highly pathogenic [4–6]. *Eimeria tenella* specifically infect epithelial cells of the caecal crypts of Lieberkhün, resulting in the induction of a range of pro- and anti-inflammatory cytokines including interleukin (IL)-6, IL-17A, IL-10 and interferon (IFN)-γ [7–11]. Infection may also result in haemorrhagic lesions of varying severity, influenced by parasite dose size and age of the bird, as well as host genotype and previous infection history [12, 13]. The presence of *Eimeria* species can also exacerbate the outcome of co-infection with bacterial pathogens such as *C*. *perfringens* (contributing to necrotic enteritis) or *Salmonella enterica* serovars Enteritidis or Typhimurium [14–16].

In the poultry industry *Eimeria* are controlled using a combination of husbandry, chemoprophylaxis and vaccination, although increasing drug resistance and consumer demand for drug-free animal produce has led to increased exploration of alternative control measures [2, 17, 18]. Pre- and probiotics have been proposed as alternatives to improve food-animal gut health and productivity [19–21], with several publications describing potential to limit *Eimeria*-induced pathology in poultry [22–24]. Microflora resident within the gastro-intestinal tract contribute to nutrient digestion, fermentation of energy substrates, the breakdown of non-starch polysaccharides and prevention of disease by reducing or blocking pathogen colonisation or replication [25, 26]. Disruption of the enteric microflora can compromise some or all of these functions, hence the need to improve understanding of apparently healthy microbiomes and the impact that pathogen exposure or pre/probiotic supplementation has on these.

Recognition of the relevance of the enteric microflora to chicken health has prompted the application of next-generation sequencing to define microbiome structure and diversity. The caeca of poultry are major pathogen reservoirs, known to possess the largest and most diverse gut microbiota in these birds [25] dominated by the phyla Firmicutes, Bacteriodetes and Proteobacteria [27]. In chickens the caeca are a pair of elongated blind sacs containing microbial communities of similar composition [28] that vary between individuals, even in similar environments [25]. Factors such as gender, age, diet, stocking density and host-genotype all can exert a significant impact on microbial composition [29–31]. Despite the significant damage that *Eimeria* causes to the chicken gastrointestinal tract, little is known about its influence on the enteric microbiome, or whether the resident microflora play any role in modulating parasite-induced pathology. The aim of this study was to define the caecal lumen microbiome of a commercial broiler chicken line following *E*. *tenella* infection, exploring variation associated with the severity of pathology induced by exposure to a single, homogeneous parasite challenge.

## Materials and methods

### Animal ethics statement

The work described here was conducted in accordance with UK Home Office regulations under the Animals (Scientific Procedures) Act 1986 (ASPA), with protocols approved by the Royal Veterinary College Animal Welfare and Ethical Review Body (AWERB). Study birds were observed twice per day for signs of illness and/or welfare impairment and were sacrificed under Home Office licence by cervical dislocation.

### Chicken management

As part of a larger study 250 day-old, Cobb500 broiler chickens were housed in coccidia-free conditions at a stocking density of 34 kg/m^2^ (anticipated end weight). Following industry standard practice chickens were vaccinated against infectious bronchitis on day of hatch (using Nobilis IB H120, MSD Animal Health, Milton Keynes, UK). Throughout the study all chickens had access to feed and water *ad-libitum*. Birds were reared on a typical starter diet supplemented with the anticoccidial Maxiban® (Elanoco; Greenfield, Indiana, USA) until 10 days of age, followed by anticoccidial-free ‘grower’ (d11-24) and ‘finisher’ (d25-29) diets (Target Feeds Ltd, Shropshire, UK).

### Parasite propagation

Sporulated *E*. *tenella* parasites of the Houghton reference strain were propagated and maintained as described previously [32, 33].

### Experimental design

At nineteen days of age chickens were randomly assigned to either control or infected groups, with each group housed in a separate room to prevent accidental cross-infection. At 21 days of age, 25 birds (group 1) received a single inoculum of 1 ml of DNase/RNase-free water, while 225 broilers in group 2 were inoculated with 35,000 sporulated *E*. *tenella* oocysts in 1 ml of water.

### Sample collection and lesion scoring

Four and a half days (108 h) post infection all birds were culled (26 days old). Gender was assigned at autopsy by identification of gonads. For this study caeca were collected from 49 female chickens and 7 male chickens. Post-mortem, caecal tissue was assessed immediately for lesions and scored following the method described by Johnson and Reid [13] by the same experienced operator. Lesions were scored from 0 to 4: 0 (no lesions), 1 (mild lesions), 2 (moderate lesions), 3 (severe lesions), 4 (very severe lesions). Caecal contents from one caeca per bird was collected into a sterile tube and immediately flash frozen in liquid nitrogen, including 8–10 birds per lesion score category. All samples were stored at—80°C until further processing. All birds were weighed two days before infection and immediately prior to culling.

### DNA extraction and preparation

DNA was extracted from each sample of caecal contents using a QIAamp DNA Stool Kit (Qiagen, Hilden, Germany), with the following modifications. Briefly, following step 2 of the QIAamp DNA Stool Kit protocol, twice the sample volume of autoclaved Ballotini beads (0.4–0.6 mm; VWR, Bristol, UK) were added and samples were homogenized for 30 seconds at 35,000 oscillations/minute in a Mini Bead-beater 24 (Bio-Spec, Bartlesville, USA) and chilled on ice. The suspension was heated for five minutes at 95°C, vortexed for 15 seconds and centrifuged for two minutes at 10,000 × *g*. The QIAamp Stool Kit protocol was resumed from step 5, following the manufacturer’s instructions. To elute DNA, 50 μl of DNase/RNase free dH_2_O (Invitrogen, Paisley, UK) was used. Eluted DNA was treated with RNase A (35 μg/ml, ThermoFisher Scientific, Hemel Hempstead, UK) for one hour at 37°C. To control for experimental error DNA was extracted from samples in triplicate, quantified using a NanoDrop 1000 Spectrophotometer (NanoDrop Technologies, Wilmington, USA) and corresponding samples combined in a 1:1:1 ratio. Combined samples were adjusted to a concentration of 5 ng/μl by dilution in DNase/RNase free dH_2_O.

### PCR amplification and sequencing

Sequencing libraries were prepared following the Illumina 16S Metagenomic Sequencing Library Protocol (https://support.illumina.com/content/dam/illumina-support/documents/documentation/chemistry_documentation/16s/16s-metagenomic-library-prep-guide-15044223-b.pdf). Specifically the V3-V4 hypervariable regions of 16S rRNA were PCR amplified from extracted caecal DNA (forward primer: 5’ cctacgggnggcwgcag 3’ and reverse primer: 5’ gactachvgggtatctaatcc 3’). Amplicon PCR followed by index PCR, to generate unique barcode sequences at the 5’ end of each primer, were carried out along with the appropriate clean up steps. Following quality control, 55 of 58 samples were taken forward for sequencing (S1 Table). The pooled DNA library (4 nM) and PhiX control v3 (4 nM) were mixed with 0.2 N fresh NaOH (Invitrogen, Paisley, UK) and HT1 buffer to produce a final concentration of 4 pM each. The library was mixed with PhiX control v3 (20%, v/v) (Illumina, San Diego, USA) and 600 μl loaded on the MiSeq reagent cartridge for Illumina sequencing. Genomic DNA from a microbial mock community was included (HM-782D, Bei Resources, Virginia, USA) as a control.

### Sequence read processing and quality control

Read pairs were merged using FLASH (Fast Length Adjustment of Short Reads) [34]. Sequences less than 400 bp were discarded using the program Trimomatic v1.2.11 [35]. Qiime v1.9.1 (Quantitative Insights Into Microbial Ecology) was used to remove barcodes and to complete data processing. Briefly, operational taxonomic units (OTUs) were taxonomically classified via uclust [36] against the curated Greengenes database v13_8 (http://greengenes.secondgenome.com/). Taxa were further classified using the EzBioCloud database [37]. OTUs belonging to the phylum cyanobacteria were discarded [38] and caecal samples with less than 1000 sequences, in total, were removed. The final biom table, containing the raw sequences for 55 samples, was used for all further analyses.

### Data analysis

Exploratory and differential abundance was analysed in R Studio v3.3.2 [39] and Bioconductor v3.3.1 [40] using the packages Phyloseq v1.19.1 [41], DESeq2 v1.14.1 [42], ggplot2 v2.2.1 [43], plyr v1.8.4 [44] and RColorBrewer v1.1–2 [45]. DESeq2 was used to identify differentially abundant phylotypes with the P-value adjusted (padj) for multiple testing using the Benjamini-Hochberg method [46]. Alpha diversity indices (Richness: Observed, Chao1, ACE (abundance based coverage estimator); richness and evenness: Shannon, Simpson, Inverse Simpson, Fisher) were obtained using the plot_richness function of the Phyloseq package. A Kruskal-Wallis test was conducted using SPSS (IBM) to assess for statistical significance. Beta diversity was analysed in Qiime v1.9.1 following normalisation by CSS (cumulative sum scaling) [47]. The weighted (quantitative) UniFrac metric was analysed [48, 49]. The nonparametric statistical method Adonis [50] was used to compare categories in Qiime v1.9.1. Data was then visualised in a Principle Coordinates Analysis (PCoA) plot. Rarefaction curves were generated in the program Calypso [51]. In all statistical tests data with an alpha value less than 0.05 was considered significant.

## Results

### Caecal microbiota

**Fig 1:**
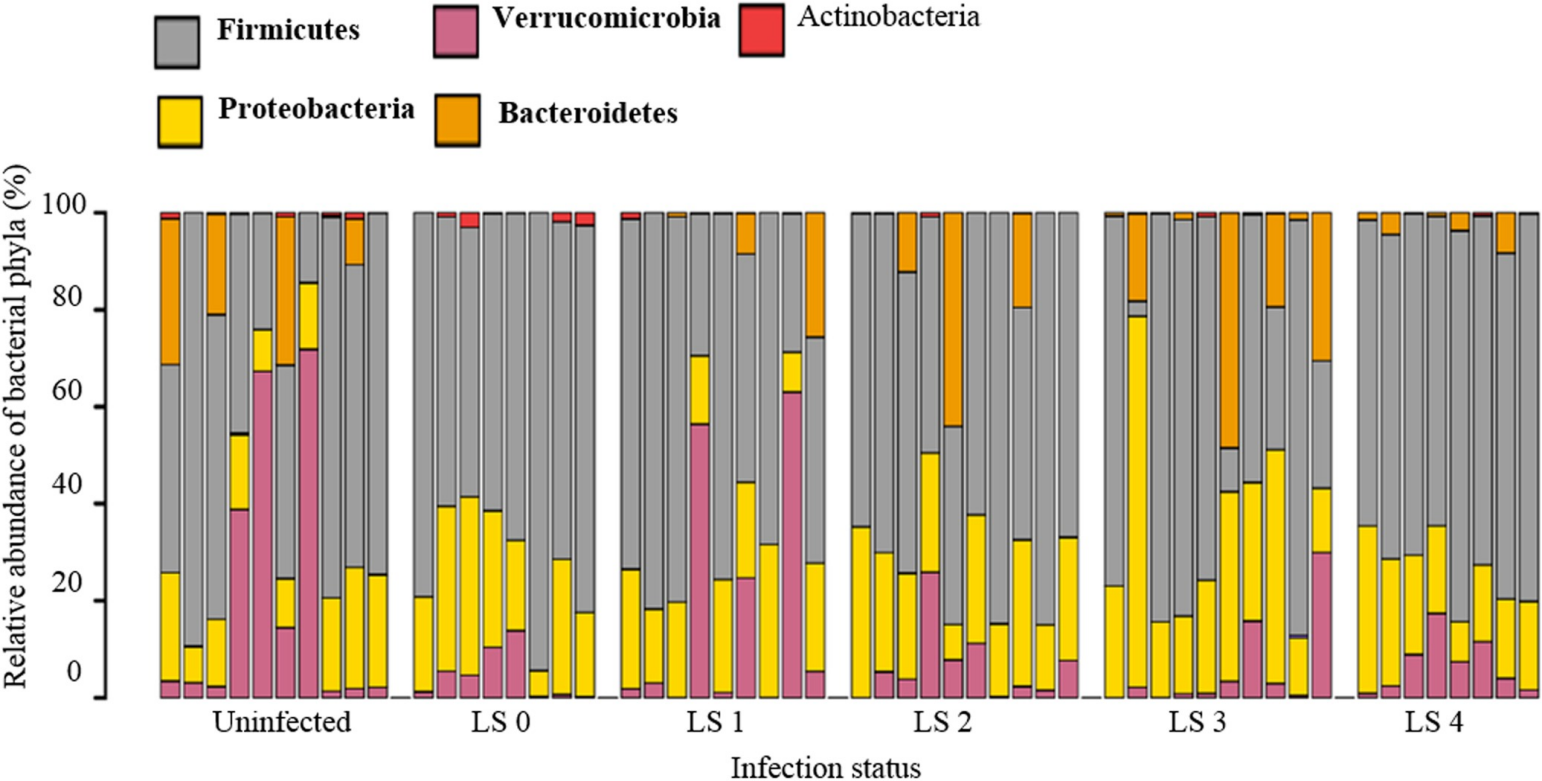
Bar chart showing relative abundance of bacterial phyla in each broiler, sorted by severity of pathology. Data were compiled using 8–10 individual caecal samples per infection status: LS 0 (n = 8, no lesions), LS 1 (n = 9, mild lesions), LS 2 (n = 10, moderate lesions), LS 3 (n = 10, severe lesions), LS 4 (n = 8, very severe lesions) and uninfected controls (n = 10). In each group there were three or four dominant phyla, as indicated in bold in the accompanying legend. In both infected (LS 0 –LS 4) and uninfected samples the phylum Firmicutes represented over 50% of all taxa in most birds.

Following quality filtering 4,858,824 sequences were obtained in total from 55 samples. The number of assembled sequences ranged from 6,742 to 249,620 per sample, with an average of 88,067 (S2 Table). All sequences have been submitted to the Sequence Read Archive and are available under the accession number SRP111033. The average assembled 16S (V3-V4) length was 448 nucleotides, ranging from 400 bp to 467 bp (S1 Fig). Rarefaction curves (S2 Fig) suggested that asymptotes were nearly reached for most samples, indicating that deeper sequencing would only reveal rare additional taxa. Using the Greengenes database these sequences were found to represent 11 bacterial phyla (Fig 1). Considerable bird to bird variation was detected, although the phyla Firmicutes, Bacteroides, Proteobacteria and Verrucomicrobia were consistently represented in both uninfected and infected groups, with Firmicutes representing over 50% of all taxa in most birds. In total 2,206 Operational Taxonomic Units (OTUs) were observed (per caecum mean 294; 115–499; S2 Table). Sequences belonging to the class *Clostridia* were found to dominate the caeca in all groups.

### Diversity of the caecal microflora

**Fig 2:**
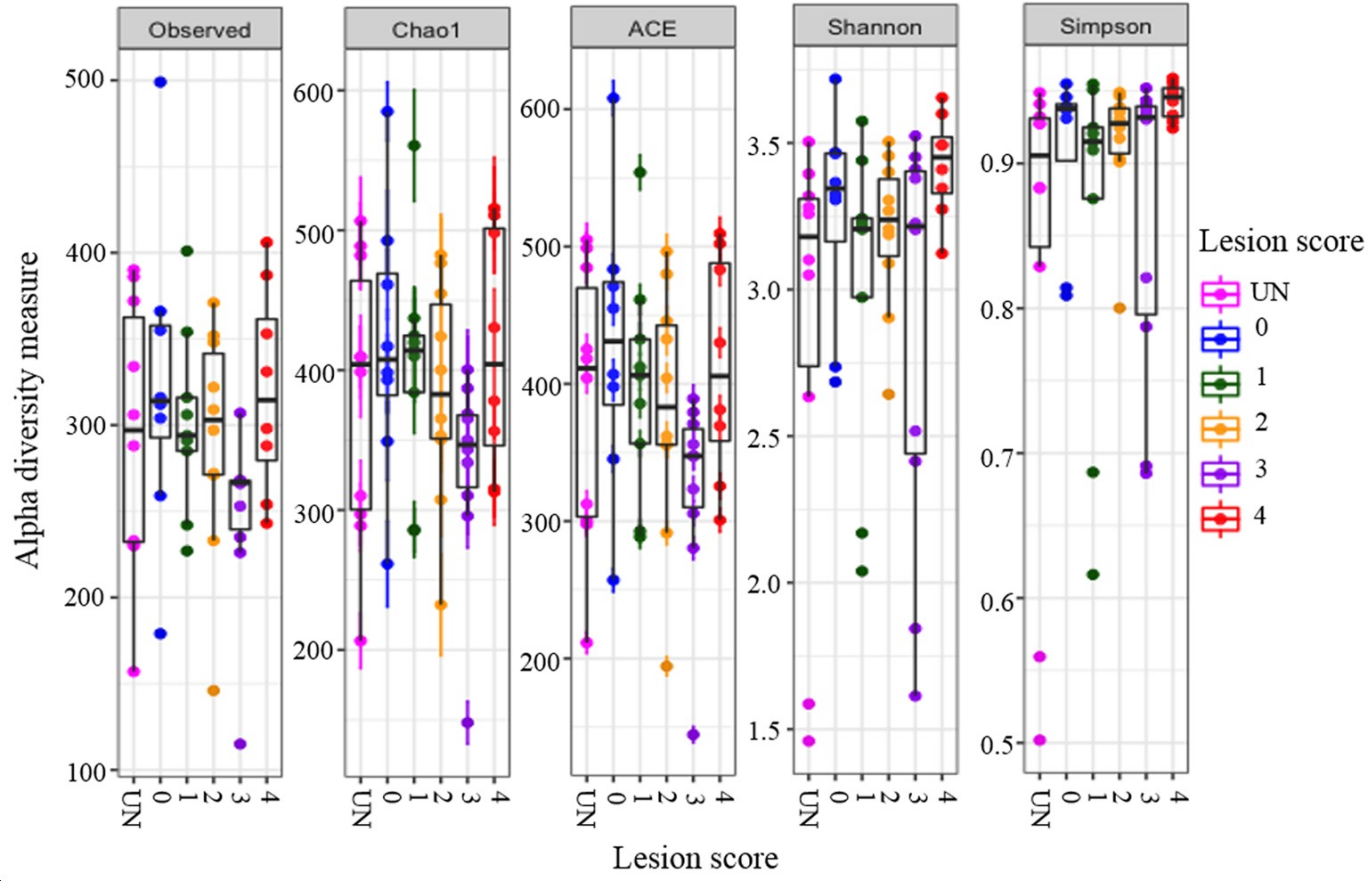
Alpha-diversity plots for each treatment group. Plot of bacterial species richness (Observed) and alpha diversity measures for each treatment group using; Chao1, ACE (Abundance-based Coverage Estimator), Shannon and Simpson tests. Circles represent individual samples, grouped by colour according to lesion score and uninfected (UN) samples. No significant differences were observed between any of the treatment groups using Kruskal-Wallis tests (P > 0.05).

Consideration of alpha diversity within the sequence datasets using the number of observed OTUs, Chao1, ACE, Shannon and Simpson indices, showed no significant variation associated with *E*. *tenella* -induced lesion score (Fig 2, S3 Table).

**Fig 3:**
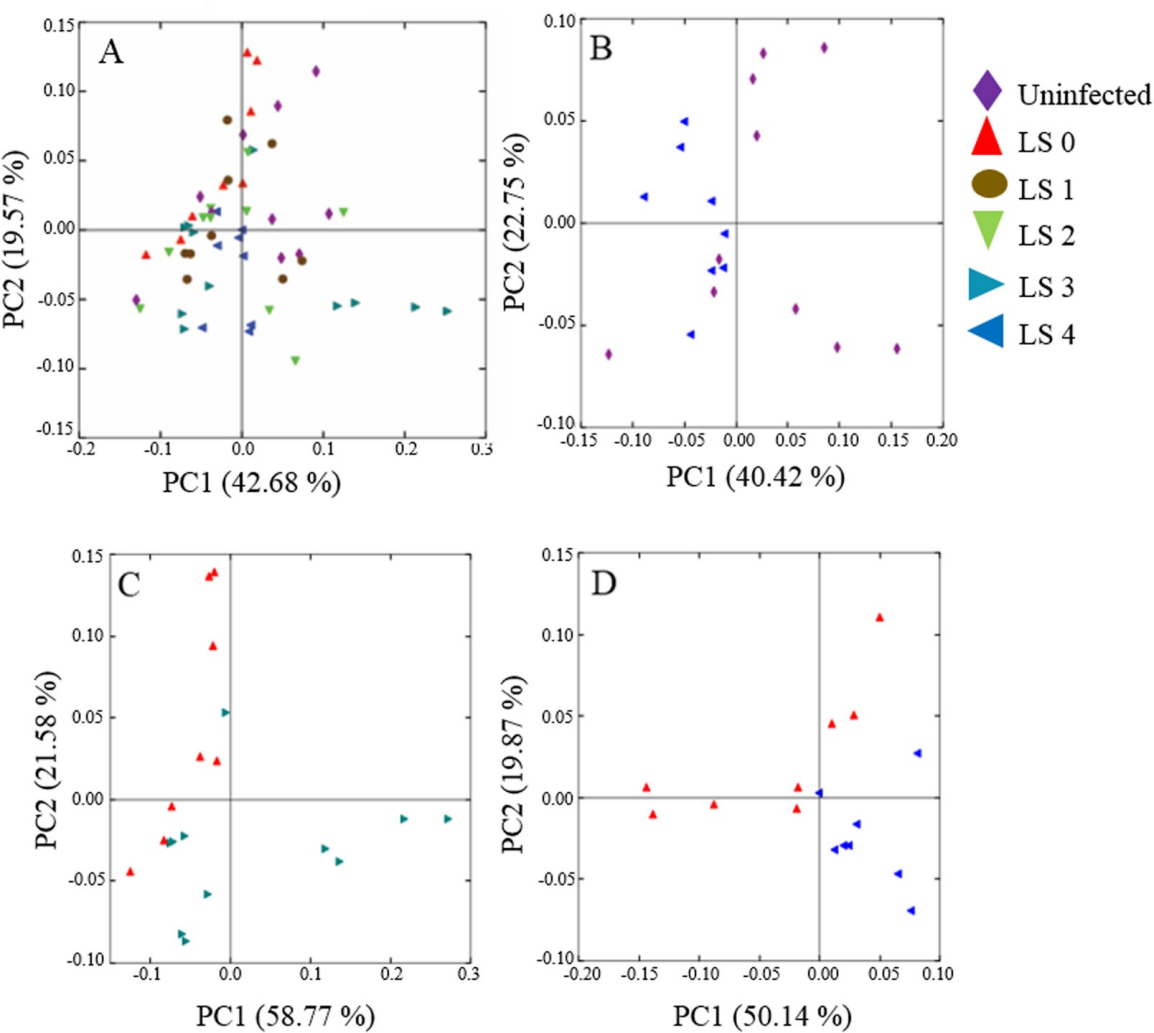
**Weighted UniFrac PCoA.** (A) All samples, LS 0 to LS 4 and uninfected (B) Uninfected *versus* lesion score (LS) 4 (C) Lesion score 0 *versus* lesion score 3 (D) Lesion score 0 *versus* lesion score 4. Each point represents a single chicken caecal microbiome. Individual groups are represented by a unique symbol and colour combination. The comparisons shown were significant according to Adonis in Qiime (P < 0.05). Lesion scores 0 to 4 indicate increasing lesion severity.

Weighted (quantitative) UniFrac was used to investigate beta diversity between lesion score groups. Analysis with Adonis revealed no statistical significance between uninfected and all infected groups (P = 0.062) but a significant relationship with individual lesion score group (Controls, LS 1–4) (R^2^ = 0.15, P = 0.025). When individual lesion score (LS) groups were compared, PCoA plots showed definitive clustering for specific comparisons (Fig 3) and significance was observed between the following groups: uninfected *versus* LS 4 (R^2^ = 0.19, P = 0.007), LS 0 *versus* LS 3 (R^2^ = 0.17, P = 0.031) and LS 0 *versus* LS 4 (R^2^ = 0.25, P = 0.004) (S4 Table). An R value equal to 1 shows the samples are completely different, while R equal to 0 means they are identical.

### Differentially abundant phylotypes

**Fig 4:**
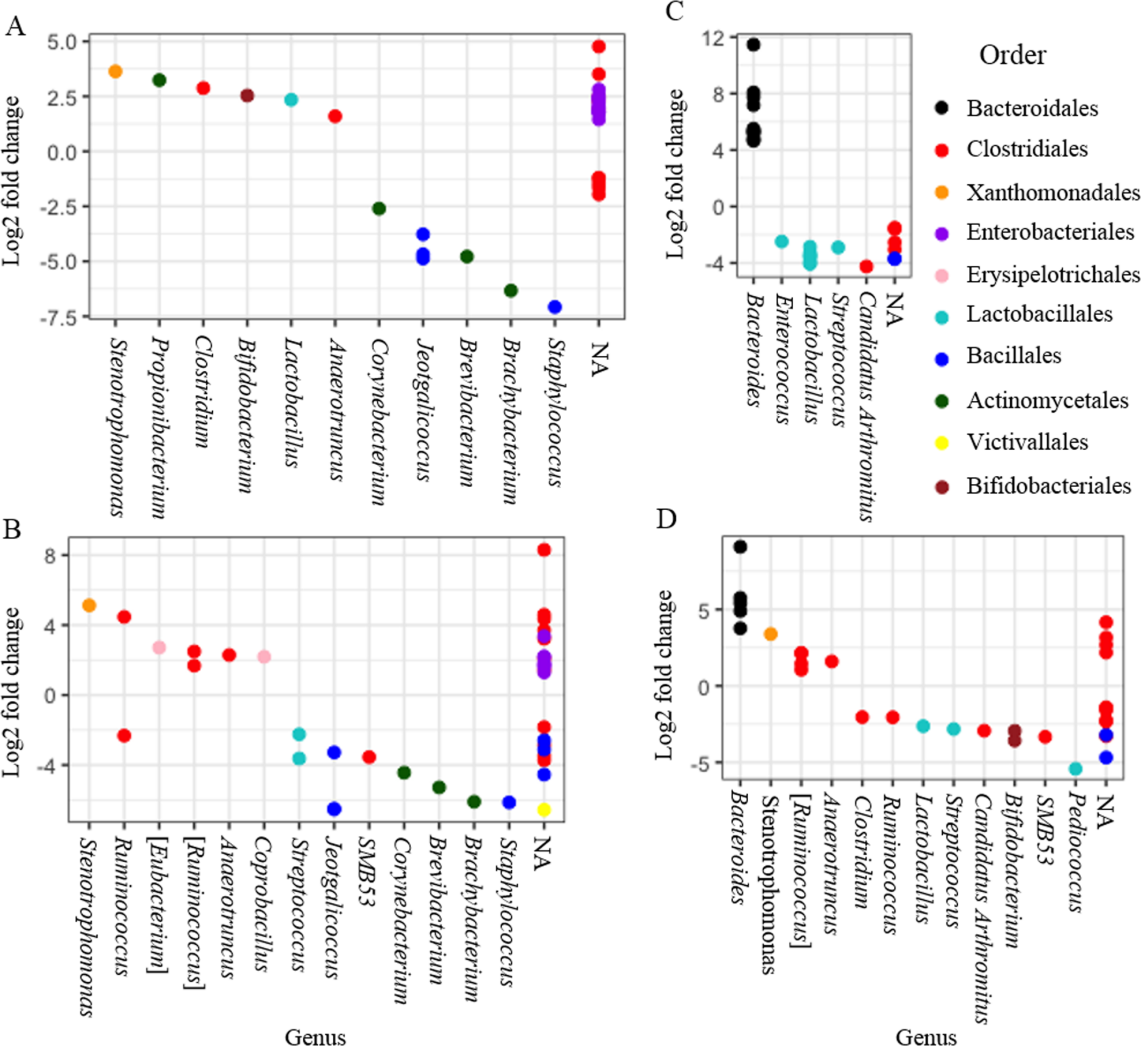
Plots of OTUs that were significantly differentially abundant (padj < 0.05) according to DESeq2 analysis. Significant OTUs are represented by single data points (with some data points overlapping), grouped by genus on the x-axis and by colour according to which taxonomic order the OTU originates. (A) Uninfected controls *versus* all infected samples (LS0 –LS 4), (B) uninfected controls *versus* lesion score 4, (C) lesion score 0 *versus* lesion score 3, (D) lesion score 0 *versus* lesion score 4.

DESeq2 was used to identify differentially abundant phylotypes following *E*. *tenella* infection. All possible comparisons were evaluated for changes in abundance (Table 1, S5–S18 Tables). In additional to individual comparisons, all samples from lesion score groups zero to four were merged to create an infected (all LS) group which was compared to the uninfected control group. All samples from lesion score groups one to four were merged to generate a symptomatic sample group, which was compared to the asymptomatic LS 0 samples (S18 Table). The number of differentially abundant OTUs was most evident when the most disparate lesion score groups were compared. The greatest number of differentially abundant OTUs was observed between the uninfected control group and the LS 4 group. Eight differentially abundant phylotypes were common across all LS groups when compared to the uninfected control group. Differentially abundant OTUs generally belonged to the following five orders: Bacillales, Clostridiales, Lactobacillales, Enterobacteriales and Bacteroidales. A graphic representation of differentially abundant OTUs for four group comparisons can be seen in Fig 4, categorised by genus and order. A full list of significant phylotypes can be found in the S5–S18 Tables.

**Table 1:** Number of significant differentially abundant OTUs identified using DESeq2 (padj < 0.05), for all group comparisons.

Lesion score groups	Uninfected	LS 0	LS 1	LS 2	LS 3	LS 4
Infected (All LS)	37					
Uninfected		26	23	16	31	41
LS 0			1	3	29	32
LS 1				0	1	10
LS 2					0	1
LS 3						0
LS 4						

Caecal samples were grouped either as uninfected or infected, with samples split according to lesion score (LS) group: 0 (no lesions), 1 (mild lesions), 2 (moderate lesions), 3 (severe lesions), 4 (very severe lesions). Each group was compared to every other group using DESeq2 in R Studio, to determine differential abundance. Comparison of all infected samples (All LS) compared to uninfected controls was also carried out. Corresponding information for all comparisons can be found in the, S5–S18 Tables.

## Discussion

The enteric microflora has been shown to play an important role in the health, welfare and productivity of commercially reared chickens [52–54]. One possible variable is infection with protozoan parasites such as the *Eimeria* species. Each *Eimeria* species present a distinct pathognomonic profile [3] and is likely to cause varied impacts in different sections of the intestine. Changes in the enteric microbiome which associate with *Eimeria* exposure may be relevant to animal welfare, food security and safety [2, 55].

Research investigating the effect that *Eimeria* has on the caecal microbiome of poultry is sparse, and to our knowledge this study is the first to specifically analyse the microbiome according to severity of caecal lesions, following exposure to a defined high dose of a single *Eimeria* species. No changes in alpha-diversity were found following infection, even in samples from birds with severe or extremely severe lesions (LS 3, LS 4). Others have also concluded that microbial community richness was not significantly affected by *E*. *tenella* [56], or mixed infection (*E*. *acervulina*, *E*. *maxima*, *E*. *tenella*) [57], but did not assess disease severity. Conversely, a combination of *Eimeria* vaccine strains (*E*. *acervulina*, *E*. *maxima*, *E*. *brunetti*, mixed suspension) induced significant changes in alpha-diversity [58] and combined *Eimeria* (mixed species)/*C*. *perfringens* infection had an even greater effect [59]. There are a number of factors that could explain the disparity in results; diet, stocking densities, host-genotype, age and gender have all been shown to influence microbiome composition [29–31, 60]. Both *Eimeria* studies reporting significant changes in alpha-diversity [58, 59] used male birds only, while the majority of birds used in the current study were female (49/56). Furthermore, the study of Boulton *et al*. from which samples in this study originate, suggest females are inherently more tolerant to *Eimeria* infection than males, as infected females had significantly more lesions than males without an associated change in body weight gain [12]. Additionally, differences in species, strain and level of *Eimeria* parasite infection may explain between study variations in alpha diversity [56].

*Eimeria* infection *per se* also did not produce a significant impact on caecal microbiome beta-diversity (P = 0.062) compared to uninfected controls. Instead, there was significant correlation between disease pathology and microbiome diversity, with the greatest differences between severely effected birds and controls. This finding raises the question of whether microbiome variation is a consequence or a potential cause of the caecal lesions. If the latter contributes, manipulation of the microbiome could have therapeutic or prophylactic benefit. These results highlight the importance of considering *Eimeria* parasite induced pathology when analysing caecal microbial diversity.

Numbers of differentially abundant taxa identified following infection were similar to previous reports investigating the influence of *Eimeria* on the caecal microbiome [58] [56]. Assessment of differential abundance was correlated to OTU/species level where possible, as it is known that analysis at higher taxonomic levels can lead to inaccuracies, however where necessary abundance at higher taxonomic levels is discussed [60]. All differential OTUs belonging to the family Enterobacteriaceae increased post-infection. Based upon analysis using EzBioCloud [37], these OTUs were most similar to either *Escherichia coli*, *E*. *fergusonii*, *Shigella flexneri* or *Shigella sonnei*. Avian Pathogenic *E*. *coli* (APEC) is of great concern within the poultry industry resulting in significant economic loss [61]. The increased abundance of OTUs associated with *Escherichia* and *Shigella* may enhance pathogenic potential, leading to opportunistic outbreaks due to immunosuppression and stress following *E*. *tenella* infection.

Following asymptomatic (LS 0) infection a relative increase in three OTUs classified as *Lactobacillus johnsonii* (EzBioCloud) was observed. More severe damage to the caeca (LS 3) resulted in a significant decrease in three different OTUs belonging to the genus *Lactobacillus*: *L*. *reuteri ×* 2 (EzBioCloud) and *L*. *pontis* (EzBioCloud). Differential taxa belonging to the genus *Lactobacillus* decreased in abundance in severe and extremely severe lesion score samples (LS 3 & LS 4) compared to those collected from asymptomatic (LS 0) chickens; these phyla were found in asymptomatic samples at similar levels to uninfected samples. Changes in abundance of *Lactobacillus* may contribute to the variation in caecal tissue damage. *Lactobacillus* based probiotics can modulate the innate and acquired immune system of poultry and have been correlated with improved outcomes in relation to bacterial and parasitic infection [23]. The anticoccidial properties of various *Lactobacilli* have been investigated with studies reporting improved body weight gain, decreased lesion scores, inhibition of cellular invasion and enhanced mucosal immunity [22, 62–64]. In conjunction with some of these anticoccidial properties, *Lactobacilli* have been shown to stimulate immune factors including IFN-γ, IL-2, IL-1β, IL-6 [22, 65] and intestinal intraepithelial lymphocytes (IEL) [62]. The elevated *Lactobacillus* in asymptomatic birds may contribute to an early immune response, reducing *E*. *tenella* invasion of epithelial cells, and mitigating development of caecal lesions. Intervention studies with various probiotic supplements have provided some support for this view [22, 64, 66]. Various probiotic formulations including PoultryStar®, Aviguard® and Broilact® have provided promising results, in laboratory testing, against a number of important poultry pathogens including *C*. *perfringens*, *S*. *enterica* serovar Enteritidis, *Campylobacter jejuni*, extended-spectrum β-lactamase producing *E*. *coli* and several *Eimeria* species parasites [24, 64, 67–72]. Furthermore, a small but significant increase in an OTU, classified as *B*. *animalis* (EzBioCloud), was observed in this study in asymptomatic samples compared to birds with extremely severe lesions (LS 4). Assessment of probiotic supplementation in chickens infected with *E*. *tenella* by Giannenas *et al*. (2012) found that *B*. *animalis* individually did not improve any of the tested parameters, however this species was included in the mixed probiotic, PoultryStar®, that showed great promise and may have synergistic properties [64].

The order Clostridiales accounted for over 50% of all taxa within the microbiome and several OTUs within this order were found to be differentially abundant following infection. Birds with the most damaged caeca (LS 4) contained the largest percentage (41.5%) of differential OTUs belonging to Clostridiales, highlighting that *E*. *tenella* induced caecal damage has a strong association with this order. Similarly, Zhou *et al*. (2017) reported the vast majority of differential OTUs (22/23) following *E*. *tenella* infection belonged to the order Clostridiales [56]. The differential OTUs belonged to several families, Lachnospiraceae, Ruminococcacea, Clostridiaceae and Peptostreptococcaceae. Classification at both genus and species levels of differential OTUs from the order Clostridiales was extremely limited and prevented detailed analysis at species level. At genus level *Clostridium* increased following infection in the all LS group, LS 0 and LS 4, conversely the genus *Ruminococcus* decreased only in LS 4 samples; similar changes were induced by mixed *Eimeria* infection in a previous study [58].

While it is unsurprising that changes in abundance of bacteria occur following *E*. *tenella* infection, of particular interest was the examination of differential taxa according to caecal lesion score. Differential OTUs were most abundant comparing samples at either end of the lesion score scale. DESeq2 analysis found 25 and 35 differential OTUs when comparing LS 0 to LS 3 and 4, respectively and ten differential OTUs between LS 1 and LS 4. Beta diversity between these groups was significant according to Adonis. Taxa belonging to the genera *Bacteroides* were among those most effected and these differential OTUs were classified as either *B*. *vulgatus* or *B*. *dorei* (EzBioCloud). Intriguingly, OTUs belonging to the genus *Bacteroides* were reduced in infected but asymptomatic chickens (LS 0) when compared with LS 3 and LS 4 samples, as well as with samples from uninfected controls. Indeed, following asymptomatic infection, OTUs belonging to the genus *Bacteroides* either completely disappeared or were present at very low levels. Overall, this genus accounted for less than 0.1% of the microflora within asymptomatic samples compared to 4.8%, 5.8%, 13.7%, 2.0% and 9.9% in LS 1, LS 2, LS 3, LS 4 and uninfected birds, respectively. Previously, *Bacteroides* were found to be abundant in the caecal microflora of chickens [28, 73] and are known to provide nutrients for the host by metabolising carbohydrates [74]. Conversely, some species of *Bacteroides* have been implicated in the pathogenesis of severe ulcerative diseases in humans and animals including ulcerative colitis and Crohn’s disease [74–77]. *Bacteroides* species have been reported to aid in the survival of some (facultative) anaerobic bacteria including *E*. *coli*, protecting against phagocytosis [78, 79]. In the most severely affected caecal samples in this study many OTUs associated with facultative anaerobes, including all differential OTUs belonging to the family Enterobacteriaceae which were increased in abundance following infection. *Bacteroides* species, as part of the commensal microflora, could protect some anaerobic bacteria in symptomatic birds, prolonging survival of pathogenic bacteria and possibly resulting in more severe tissue damage. Using DeSeq2, comparison of asymptomatic LS 0 samples to symptomatic samples, (LS1 –LS4) merged together, revealed eight OTUs belonging to the genus *Bacteroides* were decreased in asymptomatic samples while six OTUs belonging to the genus *Lactobacillus* were increased. Similar results were obtained when each individual symptomatic group was compared to LS 0 samples, with increasing numbers of differential OTUs as lesion score severity increased. These results indicate that OTUs belonging to these two genera may play a pivotal role in susceptibility or resistance to *E*. *tenella* infection. The reasons why some birds remained asymptomatic following *E*. *tenella* infection, while others were severely affected remains unknown. Host immune parameters such as the magnitude of interferon gamma and interleukin-10 responses have been implicated in the outcome of infection [12]. It is now hypothesised that a combination of factors are involved and results from this study suggest either a functional role for the enteric microbiome, or microbial variation as a consequence of infection. The importance of the enteric microbiome to fermentation and effective use of dietary resources underlines the significance of these changes [80].

## Conclusion

The current study has demonstrated that *E*. *tenella* infection of Cobb500 broilers elicited significant changes in the abundance of a number of microbial taxa in the caecal microbiome that were correlated with the most severe caecal pathology. Increases in taxa belonging to the order Enterobacteriaceae were common, as were decreases in taxa from Bacillales and Lactobacillales. These results provide new information regarding the effect that *E*. *tenella* has on the caecal microbiome of poultry and indicate the importance of accounting for differences in lesions when investigating the relationship between *Eimeria* and the poultry microbiome. A greater understanding of caecal microbiome dysbiosis associated with *Eimeria* induced caecal tissue damage could aid in the development of in-feed probiotics with the ultimate aim of reducing the most severe effects of this ubiquitous parasite. Consideration of the variation induced by infection with other *Eimeria* species is also likely to be important.

## Supporting information

S1 Fig Read length distribution. Histogram of read length, reads ranged from 400 bp to 467 bp, with an average length of 448 bp.
(DOCX)

S2 Fig Rarefaction curves of OTUs clustered at 97% sequence identity. Graph showing the number of species as a function of the number of samples for each individual sample. Samples grouped by shape and colour according to infection/lesion score (LS) status. For the majority of samples the curve is starting to become flatter to the right, indicating asymptote was reached and further sampling would yield only a few additional species.
(TIF)

S1 Table Summary of caecal samples collected and sequenced, per group. Samples were grouped by infection status and lesion score, samples were taken forward for Illumina sequencing after Bioanalyzer size verification and quality control. One mock microbial community (HM-782D, Bei resources) was included as a control.
(DOCX)

S2 Table Summary of sequenced samples. Table outlines infection status (infected or uninfected) and lesion score (LS) group: 0 (no lesions), 1 (mild lesions), 2 (moderate lesions), 3 (severe lesions), 4 (very severe lesions), total number of reads per sample, total number of OTUs* (operational taxonomic units) per sample and the sex of the chicken from which caecal samples were collected.
(DOCX)

S3 Table Comparison of alpha diversity indices across uninfected and *E. tenella* infected groups. Lesion scores (LS) 0 to 4 indicate increasing lesion severity. No statistically significant differences were observed using Kruskal-Wallis tests (P > 0.05).
(DOCX)

S4 Table Weighted UniFrac beta-diversity analysis. The Adonis method in Qiime was used to assess significance. Significant comparisons (p < 0.05) are highlighted in bold. Lesion score (LS) 0 to 4 indicate increasing lesion severity.
(DOCX)

S5 Table Differentially abundant OTUs between uninfected samples and all infected samples. (XLSX)

S6 Table Differentially abundant OTUs between uninfected samples and lesion score 0 samples. (XLSX)

S7 Table Differentially abundant OTUs between uninfected samples and lesion score 1 samples. (XLSX)

S8 Table Differentially abundant OTUs between uninfected samples and lesion score 2 samples. (XLSX)

S9 Table Differentially abundant OTUs between uninfected samples and lesion score 3 samples. (XLSX)

S10 Table Differentially abundant OTUs between uninfected samples and lesion score 4 samples. (XLSX)

S11 Table Differentially abundant OTUs between lesion score 0 and lesion score 1 samples. (XLSX)

S12 Table Differentially abundant OTUs between lesion score 0 and lesion score 2 samples. (XLSX)

S13 Table Differentially abundant OTUs between lesion score 0 and lesion score 3 samples. (XLSX)

S14 Table Differentially abundant OTUs between lesion score 0 and lesion score 4 samples. (XLSX)

S15 Table Differentially abundant OTUs between lesion score 1 and lesion score 3 samples. (XLSX)

S16 Table Differentially abundant OTUs between lesion score 1 and lesion score 4 samples. (XLSX)

S17 Table Differentially abundant OTUs between lesion score 2 and lesion score 4 samples. (XLSX)

S18 Table Differentially abundant OTUs between lesion score 0 and lesion score 1–4, symptomatic samples. (XLSX)
