# Supplementary material for: Effects of Eimeria tenella infection on chicken caecal microbiome diversity, exploring variation associated with severity of pathology
Source: PLoS One. 2017 Sep 21;12(9):e0184890. doi: 10.1371/journal.pone.0184890 (PMC5608234; doi:10.1371/journal.pone.0184890)
Supplement: S1 Fig — Histogram of read length, reads ranged from 400 bp to 467 bp, with an average length of 448 bp. (DOCX) [file pone.0184890.s001.docx]

**S1 Fig. Read length distribution**


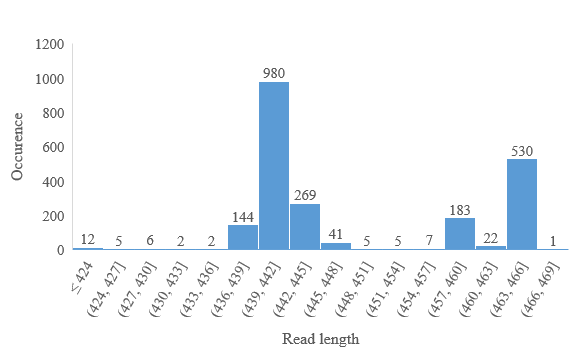


Histogram of read length, reads ranged from 400 bp to 467 bp, with an average length of 448 bp.
