# Supplementary figures and images for: Effects of Eimeria tenella infection on chicken caecal microbiome diversity, exploring variation associated with severity of pathology
Source: PLoS One. 2017 Sep 21;12(9):e0184890. doi: 10.1371/journal.pone.0184890 (PMC5608234; doi:10.1371/journal.pone.0184890)

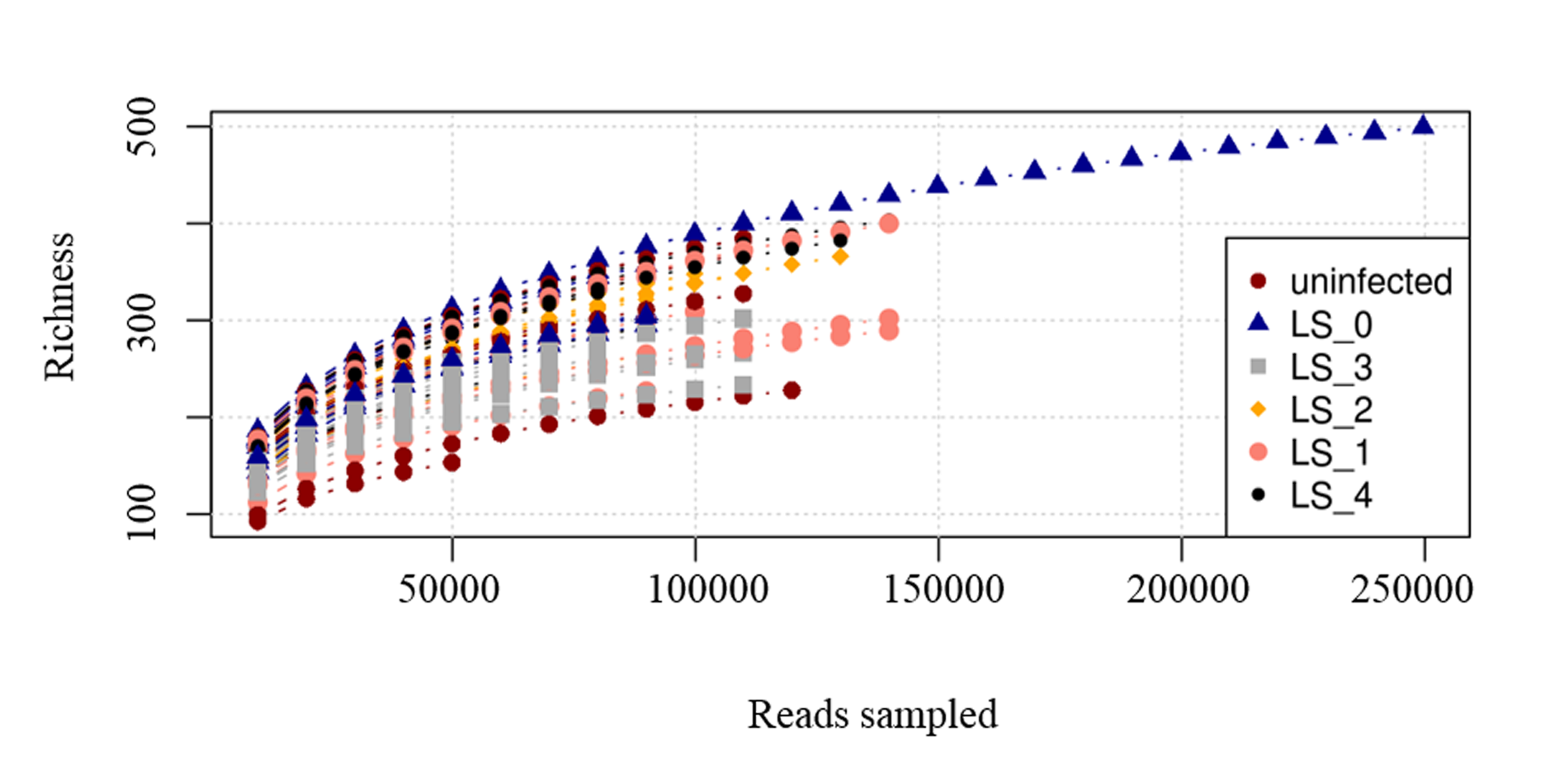

Supplement: S2 Fig — Graph showing the number of species as a function of the number of samples for each individual sample. Samples grouped by shape and colour according to infection/lesion score (LS) status. For the majority of samples the curve is starting to become flatter to the right, indicating asymptote was reached and further sampling would yield only a few additional species. (TIF) [file pone.0184890.s002.tif]
