# Supplementary material for: Effects of Eimeria tenella infection on chicken caecal microbiome diversity, exploring variation associated with severity of pathology
Source: PLoS One. 2017 Sep 21;12(9):e0184890. doi: 10.1371/journal.pone.0184890 (PMC5608234; doi:10.1371/journal.pone.0184890)
Supplement: S1 Table — Samples were grouped by infection status and lesion score, samples were taken forward for Illumina sequencing after Bioanalyzer size verification and quality control. One mock microbial community (HM-782D, Bei resources) was included as a control. (DOCX) [file pone.0184890.s003.docx]

**S1 Table. Summary of caecal samples collected and sequenced, per group.**

| Group | Number of samples collected | Number of samples Illumina sequenced |
| --- | --- | --- |
| Uninfected | 10 | 10 |
| Infected, LS 0 | 8 | 8 |
| Infected, LS 1 | 9 | 9 |
| Infected, LS 2 | 10 | 10 |
| Infected, LS 3 | 10 | 10 |
| Infected, LS 4 | 9 | 8 |
| Mock | N/A | 1 |

Samples were grouped by infection status and lesion score, and samples were taken forward for Illumina sequencing after Bioanalyzer size verification and quality control measures. One mock microbial community (HM-782D, Bei resources) was included as a control.
