# Supplementary material for: Effects of Eimeria tenella infection on chicken caecal microbiome diversity, exploring variation associated with severity of pathology
Source: PLoS One. 2017 Sep 21;12(9):e0184890. doi: 10.1371/journal.pone.0184890 (PMC5608234; doi:10.1371/journal.pone.0184890)
Supplement: S2 Table — Table outlines infection status (infected or uninfected) and lesion score (LS) group: 0 (no lesions), 1 (mild lesions), 2 (moderate lesions), 3 (severe lesions), 4 (very severe lesions), total number of reads per sample, total number of OTUs* (operational taxonomic units) per sample and the sex of the chicken from which caecal samples were collected. (DOCX) [file pone.0184890.s004.docx]

**S2 Table. Summary of sequenced samples**

| Infection status/lesion score (LS) group | Total number of reads/sample | Total number OTUs*/sample | Sex |
| --- | --- | --- | --- |
| Infected/LS 0 | 14727 | 179 | Male |
| Infected/LS 0 | 39668 | 259 | Male |
| Infected/LS 0 | 83902 | 355 | Female |
| Infected/LS 0 | 97488 | 316 | Male |
| Infected/LS 0 | 98684 | 366 | Female |
| Infected/LS 0 | 99403 | 312 | Male |
| Infected/LS 0 | 99760 | 304 | Male |
| Infected/LS 0 | 249620 | 499 | Male |
| Infected/LS 1 | 35368 | 242 | Female |
| Infected/LS 1 | 61608 | 285 | Female |
| Infected/LS 1 | 83061 | 291 | Female |
| Infected/LS 1 | 90067 | 227 | Female |
| Infected/LS 1 | 99346 | 354 | Female |
| Infected/LS 1 | 108583 | 316 | Female |
| Infected/LS 1 | 141044 | 401 | Male |
| Infected/LS 1 | 146576 | 306 | Female |
| Infected/LS 1 | 148162 | 294 | Female |
| Infected/LS 2 | 6742 | 146 | Female |
| Infected/LS 2 | 37613 | 233 | Female |
| Infected/LS 2 | 46480 | 226 | Female |
| Infected/LS 2 | 85170 | 309 | Female |
| Infected/LS 2 | 90095 | 322 | Female |
| Infected/LS 2 | 95229 | 297 | Female |
| Infected/LS 2 | 98497 | 271 | Female |
| Infected/LS 2 | 100090 | 348 | Female |
| Infected/LS 2 | 113783 | 352 | Female |
| Infected/LS 2 | 136190 | 371 | Female |
| Infected/LS 3 | 13767 | 115 | Female |
| Infected/LS 3 | 29198 | 226 | Female |
| Infected/LS 3 | 53235 | 253 | Female |
| Infected/LS 3 | 56230 | 266 | Female |
| Infected/LS 3 | 79134 | 268 | Female |
| Infected/LS 3 | 85455 | 267 | Female |
| Infected/LS 3 | 102849 | 267 | Female |
| Infected/LS 3 | 112764 | 268 | Female |
| Infected/LS 3 | 113315 | 235 | Female |
| Infected/LS 3 | 117149 | 307 | Female |
| Infected/LS 4 | 51174 | 254 | Female |
| Infected/LS 4 | 54703 | 288 | Female |
| Infected/LS 4 | 62763 | 243 | Female |
| Infected/LS 4 | 82159 | 331 | Female |
| Infected/LS 4 | 90552 | 353 | Female |
| Infected/LS 4 | 96325 | 298 | Female |
| Infected/LS 4 | 135501 | 387 | Female |
| Infected/LS 4 | 143612 | 406 | Female |
| Uninfected | 35361 | 233 | Female |
| Uninfected | 49852 | 230 | Female |
| Uninfected | 54318 | 157 | Female |
| Uninfected | 65125 | 288 | Female |
| Uninfected | 79167 | 306 | Female |
| Uninfected | 111513 | 386 | Female |
| Uninfected | 113617 | 372 | Female |
| Uninfected | 116797 | 390 | Female |
| Uninfected | 118618 | 334 | Female |
| Uninfected | 127345 | 232 | Female |

Table outlines infection status (infected or uninfected) and lesion score (LS) group: 0 (no lesions), 1 (mild lesions), 2 (moderate lesions), 3 (severe lesions), 4 (very severe lesions), total number of reads per sample, total number of OTUs* (operational taxonomic units) per sample and the sex of the chicken from which caecal samples were collected.
