# Supplementary material for: Effects of Eimeria tenella infection on chicken caecal microbiome diversity, exploring variation associated with severity of pathology
Source: PLoS One. 2017 Sep 21;12(9):e0184890. doi: 10.1371/journal.pone.0184890 (PMC5608234; doi:10.1371/journal.pone.0184890)
Supplement: S3 Table — Lesion scores (LS) 0 to 4 indicate increasing lesion severity. No statistically significant differences were observed using Kruskal-Wallis tests (P > 0.05). (DOCX) [file pone.0184890.s005.docx]

**S3 Table. Comparison of alpha diversity indices across uninfected and E. tenella infected groups.**

| Alpha diversity indexes | Lesion score status | | | | | |
| --- | --- | --- | --- | --- | --- | --- |
|  | Uninfected | LS 0 | LS 1 | LS 2 | LS 3 | LS 4 |
| Observed (OTUs) | 834 | 815 | 767 | 801 | 732 | 809 |
| Chao1 | 1109 | 986 | 956 | 988 | 891 | 1025 |
| ACE | 1051 | 974 | 938 | 981 | 906 | 991 |
| Shannon | 3.4 | 3.8 | 3.5 | 3.7 | 3.5 | 3.7 |
| Simpson | 0.93 | 0.96 | 0.94 | 0.96 | 0.94 | 0.96 |

Lesion scores (LS) 0 to 4 indicate increasing lesion severity. No statistically significant differences were observed using Kruskal-Wallis tests (P > 0.05).
