# Supplementary material for: Effects of Eimeria tenella infection on chicken caecal microbiome diversity, exploring variation associated with severity of pathology
Source: PLoS One. 2017 Sep 21;12(9):e0184890. doi: 10.1371/journal.pone.0184890 (PMC5608234; doi:10.1371/journal.pone.0184890)
Supplement: S4 Table — The Adonis method in Qiime was used to assess significance. Significant comparisons (p < 0.05) are highlighted in bold. Lesion score (LS) 0 to 4 indicate increasing lesion severity. (DOCX) [file pone.0184890.s006.docx]

**S4 Table. Weighted UniFrac beta-diversity analysis.**

| Groups compared | P value (Adonis) | R^2^ (Adonis) |
| --- | --- | --- |
| Uninfected vs infected (all LS)  **All LS groups (inc uninfected)**  **Asymptomatic (LS 0) vs symptomatic (LS 1 – LS 4)** | 0.062  **0.025**  **0.027** | 0.04  **0.15**  **0.05** |
| Uninfected vs LS 0 | 0.091 | 0.11 |
| Uninfected vs LS 1 | 0.147 | 0.09 |
| Uninfected vs LS 2 | 0.093 | 0.10 |
| Uninfected vs LS 3 | 0.063 | 0.11 |
| **Uninfected vs LS 4** | **0.007** | **0.19** |
| LS0 vs LS1 | 0.166 | 0.09 |
| LS0 vs LS2 | 0.058 | 0.13 |
| **LS0 vs LS3** | **0.031** | **0.17** |
| **LS0 vs LS4** | **0.004** | **0.25** |
| LS1 vs LS2 | 0.783 | 0.02 |
| LS1 vs LS3 | 0.184 | 0.08 |
| LS1 vs LS4 | 0.148 | 0.09 |
| LS2 vs LS3 | 0.324 | 0.05 |
| LS2 vs LS4 | 0.443 | 0.05 |
| LS3 vs LS4 | 0.25 | 0.06 |

The Adonis method in Qiime was used to assess significance. Significant comparisons (p < 0.05) are highlighted in bold. Lesion score (LS) 0 to 4 indicate increasing lesion severity.
